# Predictive Modeling of *In Vivo* Response to Gemcitabine in Pancreatic Cancer

**DOI:** 10.1371/journal.pcbi.1003231

**Published:** 2013-09-19

**Authors:** James J. Lee, Justin Huang, Christopher G. England, Lacey R. McNally, Hermann B. Frieboes

**Affiliations:** 1 School of Medicine, University of Louisville, Louisville, Kentucky, United States of America; 2 Department of Pharmacology/Toxicology, University of Louisville, Louisville, Kentucky, United States of America; 3 James Graham Brown Cancer Center, University of Louisville, Louisville, Kentucky, United States of America; 4 Department of Bioengineering, University of Louisville, Louisville, Kentucky, United States of America; University of Notre Dame, United States of America

## Abstract

A clear contradiction exists between cytotoxic *in-vitro* studies demonstrating effectiveness of Gemcitabine to curtail pancreatic cancer and *in-vivo* studies failing to show Gemcitabine as an effective treatment. The outcome of chemotherapy in metastatic stages, where surgery is no longer viable, shows a 5-year survival <5%. It is apparent that *in-vitro* experiments, no matter how well designed, may fail to adequately represent the complex *in-vivo* microenvironmental and phenotypic characteristics of the cancer, including cell proliferation and apoptosis. We evaluate *in-vitro* cytotoxic data as an indicator of *in-vivo* treatment success using a mathematical model of tumor growth based on a dimensionless formulation describing tumor biology. Inputs to the model are obtained under optimal drug exposure conditions *in-vitro*. The model incorporates heterogeneous cell proliferation and death caused by spatial diffusion gradients of oxygen/nutrients due to inefficient vascularization and abundant stroma, and thus is able to simulate the effect of the microenvironment as a barrier to effective nutrient and drug delivery. Analysis of the mathematical model indicates the pancreatic tumors to be mostly resistant to Gemcitabine treatment *in-vivo*. The model results are confirmed with experiments in live mice, which indicate uninhibited tumor proliferation and metastasis with Gemcitabine treatment. By extracting mathematical model parameter values for proliferation and death from monolayer *in-vitro* cytotoxicity experiments with pancreatic cancer cells, and simulating the effects of spatial diffusion, we use the model to predict the drug response *in-vivo*, beyond what would have been expected from sole consideration of the cancer intrinsic resistance. We conclude that this integrated experimental/computational approach may enhance understanding of pancreatic cancer behavior and its response to various chemotherapies, and, further, that such an approach could predict resistance based on pharmacokinetic measurements with the goal to maximize effective treatment strategies.

## Author Summary

There are few treatment options for advanced pancreatic cancer. The chemotherapeutic drug Gemcitabine is routinely used, yet 95% of patients die within 5 years of diagnosis. Surprisingly, Gemcitabine experiments with pancreatic tumor cells in the laboratory dish show that most cells will be killed by this drug. It is obvious that the dish does not adequately represent the more complex condition in real tumors. We apply mathematical modeling to simulate tumor growth to try to understand how results from the laboratory could be used to predict the treatment response in real tumors. The model simulates flow of substances such as oxygen within tumors and how this flow affects the response of cells to drug treatment. We set the inputs for the model with values obtained from the laboratory experiments. The model predicts the treatment to mostly fail in real tumors regardless of the characteristics of individual cells. We confirm these results by treating real tumors in mice, showing that our integrated experimental/computational approach may improve the understanding of pancreatic cancer behavior and response to chemotherapy, and also help to optimize treatment strategies.

## Introduction

We aim to quantify the link between pancreatic tumor growth observed *in-vitro* and that observed *in-vivo* by providing a novel integrated experimental/computational approach to predict the cancer drug response. The most common chemotherapy drug, Difluorodeoxycytidine (dFdC, or gemcitabine), is a cytidine analogue which has shown activity as a single agent against solid human tumors. Multiple studies have evaluated the efficacy of gemcitabine in the treatment of unresectable and metastatic pancreatic cancer. However, the success of gemcitabine to treat pancreatic cancer is limited, resulting only in a slight prolongation of survival and a moderate improvement in quality of life. An early study in advanced pancreatic cancer showed a measurable response in 23.8% of patients with median survival of 5.7 months and 18% survival at 12 months [1]. Combination therapies including gemcitabine have been associated with minimal improvement when compared to gemcitabine alone [2]–[5].

Laboratory studies have demonstrated the *in-vitro* efficacy of treatment strategies employing gemcitabine, but have failed to confirm the effectiveness of these strategies *in-vivo* using orthotopic pancreatic adenocarcinoma mouse models [6]–[7]. *In-vitro* conditions provide cells with unlimited access to oxygen, nutrients and drug, and lack interactions present in 3D tissue with the extracellular matrix and with host cells. The *in-vivo* parameters of intercellular and extracellular contributors to drug response are poorly understood because these parameters are difficult to measure in living tissue. Insufficient vasculature within pancreatic tumors creates a hypoxic, nutrient-deficient, and toxic environment due to the impaired blood flow and accumulation of metabolites [8]–[10]. Further, these hostile conditions select for cells that can survive with less than normal access to oxygen, nutrient, and pH conditions. Stressed tumor and host cells release a net balance of pro-angiogenic growth factors to induce neovascularization; by the time a pancreatic tumor reaches a clinically detectable size, it is usually in the vascular growth phase and contains highly aggressive cell species. Using a mouse model of pancreatic adenocarcinoma, Tuveson and coworkers recently reported that drug is indeed inefficiently delivered to pancreatic tumors because of deficient vasculature and abundant stromal content [11]–[12].

The contradictory *in-vitro* and *in-vivo* observations illustrate the critical need for biologically realistic and predictive mathematical models that can integrate information about cell proliferation and death with microvascular deficiency and diffusion gradients in the microenvironment. Although experimental studies have revealed a wealth of insight into molecular mechanisms of intrinsic resistance to gemcitabine in pancreatic cancer [13] and have helped to elucidate the critical role of the stroma [11]–[12], [14]–[15], there is a paucity of mathematical models to quantitatively evaluate the growth of pancreatic tumors and their treatment response. It was noted almost 50 years ago that tumor growth in 3D spatial dimensions could not be satisfactorily modeled by simple exponential formulations [16], and that this growth could be better described if fit to a Gompertzian model [17] – a fact confirmed experimentally even with 3D cell cultures *in-vitro* (e.g., [18]). Recently, Iacobuzio-Donahue and coworkers provided a quantitative analysis of the timing of the genetic evolution of pancreatic cancer, showing that it takes at least a decade from tumorigenesis initiation until the emergence of a parental clone, followed by ∼6.8 years until the emergence of cells capable of surviving metastasis, and then followed by an additional ∼2.7 years until the patient's death [19]. Michor and coworkers analyzed the effects of different treatment modalities for pancreatic cancer using a stochastic model, finding that restraining the tumor cell growth earlier during treatment may yield better outcomes than tumor resection [20].

Here, we study pancreatic tumor growth and treatment response by applying the nonlinear model advanced by Cristini et al [21] and further developed in [18], [22]–[24], which enables description of tumor growth through a set of two dimensionless parameters that relate to mitosis rate, apoptosis rate, cell mobility, and cell adhesion. This model builds upon a formulation of previous continuum models [25]–[27] that describe conservation laws for concentrations of oxygen/nutrients and cells. We perform experiments to measure response to gemcitabine for MiaPaca-2 and S2-VP10 cells grown *in-vitro*, and obtain input parameter values for the mathematical model. We then apply the model, taking into account the effects of diffusion gradients *in-vivo*, to predict the response with real tumors in live mice. The results provide a quantitative measure of the extent through which the 3D microenvironment, including deficient vascularization and diffusion gradients, may affect the drug response of pancreatic tumors beyond considerations of intrinsic resistance. In this manner we develop an integrated experimental/computational approach to theoretically predict the response of pancreatic cancer to drug treatment *in-vivo* given input from *in-vitro* experiments.

## Methods

We performed experiments with pancreatic cancer cells *in-vitro* to measure proliferation and apoptosis. Cancer cells were also injected into the pancreas of live mice to grow tumors *in-vivo*. The extent of tumor vascularization was assessed from these live tumors. The experimental measurements were used to set the mathematical model parameters to calculate the tumor growth, and the simulation results were then compared to the *in-vivo* tumor observations.

### Experimental Model

Experiments used pancreatic cancer S2-VP10 cells (generous gift from Dr.M.Hollingsworth, University of Nebraska [28]) to represent aggressive tumors and MiaPaCa-2 (ATCC) cells to represent less aggressive tumors. Cells were maintained under standard cell culture conditions. Gemcitabine (Eli Lilly) was used as the cytotoxic drug. Details of experimental methods are given below.

#### Cell viability *in-vitro*

Cell viability (as percentage of untreated control +SE) was measured as described previously [29] using ATPlite (PerkinElmer) assay. Briefly, cells incubated overnight with 2,000 cells per well in 96-well plates were treated with 100 µl of gemcitabine (0, 3, 30, 300 nM) for 24 hours (*n* = 4). Luciferase expressing clones, S2-VP10L and MiaPaCa2-B, were established by transducing cells with a non-replicating retrovirus containing firefly luciferase gene (Stratagene). Single cell clones were isolated and bioluminescence signal was confirmed using IVIS 100 system (Caliper, Hopkinton, MA). Growth rates and drug response of parental cells and clones were identical (data not shown).

#### Orthotopic model *in-vivo*

Female severe combined immunodeficient (SCID) mice 6 weeks of age (Harlan Laboratories) were allowed to acclimate for 1 week before implantation. Strict adherence to the University of Louisville Institutional Animal Care and Use Committee (IACUC 11110) approved protocol was maintained. Orthotopic cell implantation followed procedure in [30]. Briefly, mice were anesthetized (isoflurane gas), incised (1-cm) in left upper abdomen quadrant, and the pancreas was exposed by retraction of the spleen. 2.5×10^6^ MiaPaCa-2B (1×10^5^ S2-VP10L) cells in 30 µL of DMEM were injected into the tail of the pancreas. A sterile cotton tipped applicator was used to cover the injection site for 30 s to prevent peritoneal leakage. The spleen was returned to the appropriate position in the abdomen, and the skin and peritoneum were closed in one layer with three interrupted 5-0 Prolene sutures.

#### Tumor growth inhibition and survival studies

Bioluminescence imaging was used to assess orthotopic implantation immediately following surgery (Advanced Molecular Imager AMI-1000-X, Spectral Instruments Imaging). Mice with detectable leakage from the pancreas were removed from the study. Mice were injected *i.p.* with 100 µl of Luciferin (2.5 mg) to undergo bioluminescence imaging. Region of interest (ROI) analysis was used to measure light emitted using AMIVIEW software. Mice were sorted into groups of equal mean bioluminescent signal. Mice were treated when tumors reached ∼1.5 mm radii; while mice implanted with S2VP10L cells were treated beginning 7 days after injection, mice with MiaPaCa-2B cells were treated beginning 14 days after injection. Treatment groups (*n* = 8/group) included saline or 50 mg/kg gemcitabine administered *i.p.* weekly. Each mouse was imaged weekly to monitor tumor growth and metastasis. Treatment efficacy was assessed using bioluminescence imaging and analyzed using ANOVA with a p-value threshold of 0.05. A sample set of bioluminescent emission data for untreated mice at day 7 post-orthotopic injection of S2-VP10 tumor cells is summarized in **Table S1**.

### Mathematical Model of Tumor Growth

We model the growth of pancreatic tumors *in vivo* building upon the formulation first described in [21] and further developed in [18], [22]–[24]. This modeling generally describes tumor-related variables as continuous fields by means of partial differential equations (PDE) [23]. Tumors are treated as a collection of tissue, described by densities or cell volume fractions. Individual cells and other elements are not tracked. Model variables include the cell volume fractions and concentrations of cell substrates such as oxygen and nutrients diffusing from the capillaries. The vasculature itself is not represented but only the diffusion of substances from it. The equations are solved using numerical solvers [23].

The **Supplement** contains a detailed summary of the model formulation and underlying assumptions. Briefly, cells are represented as a continuous domain in 3D space and receive substrates (oxygen, nutrients) via diffusion from vasculature within the tissue. Following classical continuum tumor models by [25]–[27] and others, it may be assumed that the cell density is constant in the proliferating tumor domain; hence, mass changes correspond to volume changes. The tumor is treated as an incompressible fluid and the tissue elasticity is neglected. Cell-to-cell adhesive forces are modeled by a surface tension at the tumor-tissue interface [26]. The growth of the mass is governed by the balance between cell proliferation and apoptosis, which includes the drug effect. The rate of mitosis depends on the concentration of cell substrates (oxygen, glucose), which obey diffusion-reaction equations in the tumor volume. The bulk source of the cell substrates and drug is in the vasculature.

#### Diffusible substances

The diffusion of cell substrates and drug in a solid tumor, combined with cellular uptake, creates and maintains gradients of these substances through the 3D tumor tissue, assumed to be fairly compact. In particular, pancreatic tumors are typically insufficiently vascularized and with a denser stroma, which would be reflected in the cell proliferation and apoptosis values dependent on the availability of cell substrates and drug in the tumor microenvironment. We assume that the capillary concentration within the tumor is uniform but insufficient to adequately support all of the tumor cells, hence maintaining gradients of diffusible substances (Eq. 1 below). This contrasts with healthy tissue in which normal cell proliferation and apoptosis balanced with the availability of oxygen and nutrients from the vasculature avoids the formation of such gradients.

The principle of conservation of mass is applied to account for these diffusible substances within the tumor domain Ω(*t*) [21], [23]: the net rate Γ of cell substrates diffusing across tumor viable regions minus the amount uptaken by tumor cells in these regions equals the rate of change of the substrates (at steady state, this rate is 0). That is,
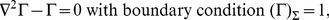
(1)where the amount diffusing in space (first term) balances the cellular uptake (second term).

The boundary between tumor and healthy tissue is Σ(*t*), **n** is the unit outward normal to Σ, *t* is time. Space and time are normalized with *L_D_* (diffusion distance of oxygen) and 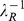 (tissue relaxation rate), respectively. At the boundary, the tissue is assumed to be normally vascularized (at maximum and uniform oxygen/nutrients) [21], [23].

#### Tumor pressure

The change in tissue pressure balances within the tumor tissue domain. Denoting **x** as position in 3D space [21], [23], we have:
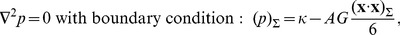
(2)where at the tumor boundary, the tissue pressure depends on local total curvature *κ* and the dimensionless parameters *A* and *G* (below).

#### Tumor velocity

The tumor growth depends on cell proliferation and death, and extent of vascularization. The resulting tumor tissue velocity depends on the change in tissue pressure, nutrient availability, and location of proliferating and dying cells within the tumor [21]:
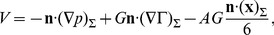
(3)where the velocity *V* of the tumor boundary depends on the change in pressure *p* (second term), rate of change in cell substrates Γ (third term), and position in space **x** (fourth term) [21]. The tumor is growing when *V*>0, unchanging when *V* = 0, and shrinking otherwise.

The non-dimensional parameter
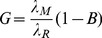
(4)describes the relative rate of cell mitosis *λ_M_* to relaxation *λ_R_*; this relaxation depends on cell mobility and cell-to-cell adhesion [21]. The non-dimensional parameter
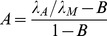
(5)describes the balance between cell apoptosis *λ_A_* and mitosis *λ_M_* [21]. Both parameters *A* and *G* include the extent of vascularization through the non-dimensional parameter *B*:
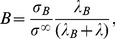
(6)where *λ* is nutrient uptake rate by tumor cells, *λ_B_* is vasculature-tissue nutrient transfer rate, *σ_B_* is nutrient concentration in the vasculature, and *σ^∞^* is nutrient concentration outside the tumor volume (i.e., in the host tissue) [21], [23].

#### Tumor size

As a first approximation, the bulk tumor shape is approximated by a sphere (as was observed experimentally). The evolution of the tumor radius *R* then depends mainly on the ratio of cell death to mitosis (parameter *A*) and ratio of mitosis to relaxation (parameter *G*):
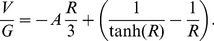
(7)This equation describes spherically-symmetric tumor growth using **Eq. (3)** [21]. The tumor radius *R* is non-dimensionalized by *L_D_* (diffusion distance of oxygen).

Three tumor growth regimes can be identified from **Eq. (7)** (as described in [21]–[23]):

1. *Low vascularization*: *A*>0 and *G*≥0 (i.e., *B*<*λ_A_*/*λ_M_*). Tumor evolution is monotonic and leads to a stationary radius *R_∞_*, i.e., the growth is arrested primarily by lack of oxygen/nutrients in the microenvironment.
2. *Moderate vascularization*: *A*≤0 and G≥0 (i.e., *λ_A_*/*λ_M_*≤*B*<1). Tumors grow unbounded, with growth that tends to linear for *A* = 0 (with velocity *V*→*G* as R→*∞*) and to exponential for *A*<0 (with velocity *V*→*AGR*/3 as R→*∞*).
3. *High vascularization*: *G*<0 (i.e., *B*>0). Depending on initial tumor radius size, unbounded growth may occur for *A*>0; otherwise, tumor will shrink and die as would also occur for *A*<0 (i.e., domination of cell apoptosis, *B*<*λ_A_*/*λ_M_*).

## Results

### Cytotoxicity *In-Vitro*

**Figure 1:**
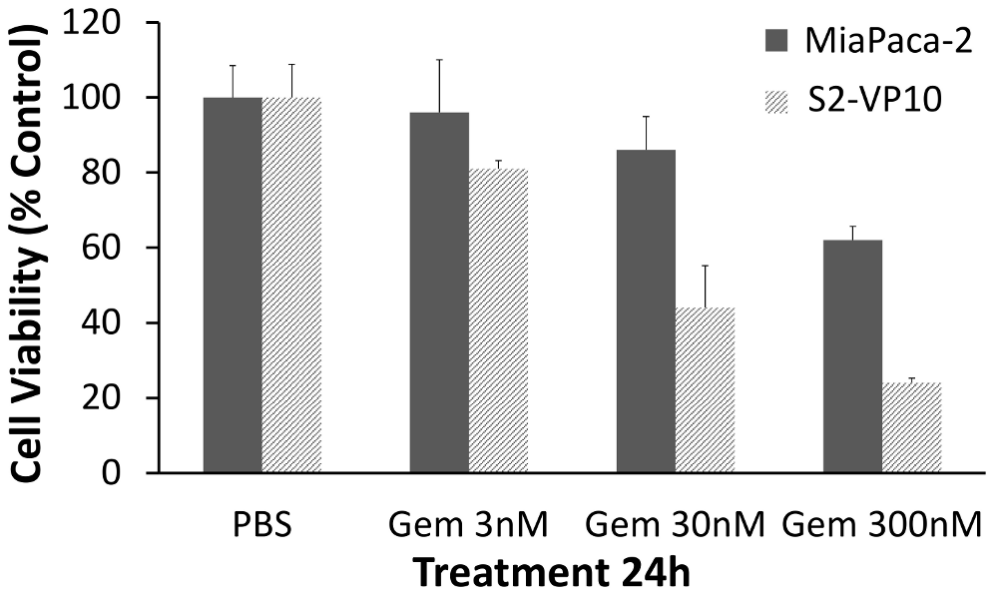
Gemcitabine toxicity *in-vitro* for MiaPaCa-2 and S2-VP10 pancreatic cell lines. Viability for cells treated with varying concentrations of Gemcitabine was determined after 24 hours of drug exposure.

Gemcitabine-induced cytotoxicity varied between MiaPaCa-2 and S2-VP10 cells (**Figure 1**), with S2-VP10 cells showing higher sensitivity. At 24 hours, MiaPaCa-2 cells were moderately resistant with cell viability (percent of control) at 62% when treated with 300 nmol/L. At 24 hours, S2-VP10 cells were more responsive as 24% of cells were viable after treatment with this dosage. Assuming a mouse weight ∼20 g and plasma volume of ∼1.2 ml [31], *in-vivo* bolus concentrations corresponding to the *in-vitro* dosages are 2.82×10^−3^, 2.82×10^−2^, and 2.82×10^−1^ mg Gemcitabine/kg mouse, respectively.

### Mathematical Model of *In-Vivo* Response

The relative strength of apoptosis *A* is the main free parameter in the equation describing the tumor growth (**Eq. 7**). This parameter, in turn, depends on the rates of cell apoptosis *λ_A_* and mitosis *λ_M_* (**Eq. 5**) and the extent of vascularization *B* (**Eq. 6**), all of which are calculated from the experimental measurements as described below. The model parameters and their associated biological meaning are summarized in **Table S2**.

Parameter *B* (representing extent of vascularization), is estimated from microvessel density (MVD) in immunohistochemistry slides. We assume that tumor growth is associated with a bulk source of oxygen/nutrients, and, hence, the values of parameters *λ*, *λ_B_*, *σ_B_*, and *σ^∞^* (**Eqs.3** **–** **6**) are assumed to be uniform for cells under similar conditions. This implies that growth is modeled to be limited by the diffusion of cell substrates [21] through the tumor (**Eq.3**). Assuming *σ^∞^* is uniform (i.e., surrounding host tissue is well-vascularized), the nutrient concentration is also assumed constant outside the tumor and at the tumor-host interface (Σ).

**Figure 2:**
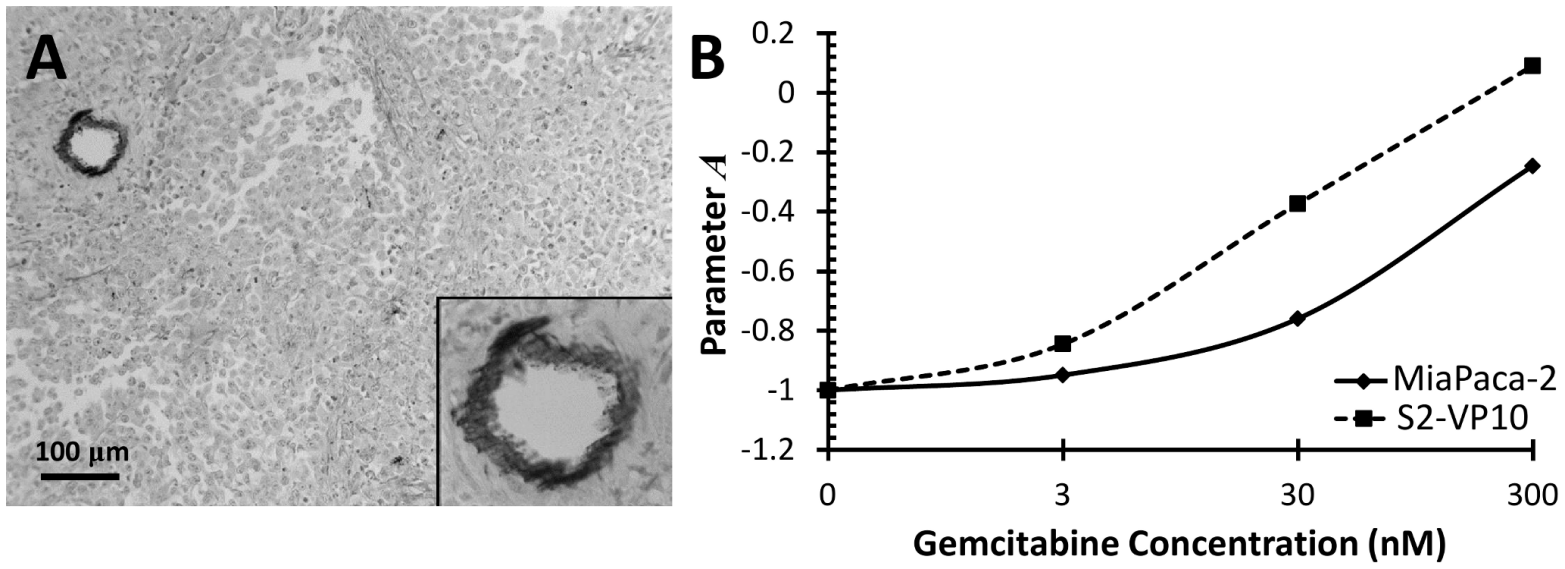
Calculation of MVD and Parameter *A*. (**A**) Sample S2-VP10 histology slide stained for Factor VIII (40×). (**B**) Parameter *A* (non-dimensional) describing the relative strength of apoptosis calculated from *in-vitro* measurements as a function of gemcitabine concentration (and with extent of vascularization parameter *B* = 0.43).

We estimate the extent of vascularization in normal tissue using PO_2_ as a critical cell substrate, which is ∼40 mmHg in capillaries and drops to ∼8 mmHg entering the tissue [32]. Therefore, in **Eq.6** for parameter *B*, *σ_B_* = 40 mmHg and *σ^∞^* = 8 mmHg. Assuming that the rate of oxygen/nutrient supply balances the uptake in the tissue, the ratio of these values is 0.5, and thus in normal tissue *B*∼2.5. To estimate *B* in tumor tissue, we calculate the ratio between tumor and normal tissue MVD. Pancreatic histology (**Figure 2A**) was used to estimate MVD values (**Supplement**), 1.56±0.94% and 0.27±0.26% in normal and tumor tissue, respectively, yielding a ratio of 0.17. Scaling *B* in normal tissue by the MVD ratio yields a value of *B*∼0.43 in tumor tissue.

Parameter *A*, representing the ratio of cell death (*λ_A_*) to mitosis (*λ_M_*) (**Eq.5**), is calculated by evaluating mitosis and death from the *in-vitro* cytotoxicity data. We link rates *λ_M_* and *λ_A_* to the experimental measurements [33]: 
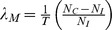
 and 
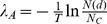
, where at time *T* (day), viable cell counts are *N_C_* (control), *N_I_* (initial), and *N*(*d*) (treated). With *T* = 1day, mitosis rate *λ_M_* was ∼1.23 days^−1^ and ∼2.63 days^−1^ for MiaPaCa-2and S2-VP10 cells, respectively. Apoptosis rate *λ_A_* was calculated at each gemcitabine concentration (**Table 1**). As a first approximation, we assume these rates are invariant for cells under similar conditions and during the time of treatment [18], [21]–[23], [26], [33], thus simulating an optimal course of treatment. Parameter *A* is then calculated with the estimated value of *B* as a function of gemcitabine concentration (**Figure 2B**). Based on the classification of the three possible regimes of tumor vascularization by the model (**Methods** **, Section 2.4**), the *in-vivo* tumors were designated as mostly moderately vascularized (**Table 1**).

**Table 1:** Calculated values of cell apoptosis *λ_A_* and model parameter *A* (using **Eq.5**) at various gemcitabine concentrations.

Gem	MiaPaCa-2				S2-VP10			
(nM)	*λ_A_* **(day^−1^)**	*λ_A_*/*λ_M_*	*A*	Vascularization	*λ_A_* **(day^−1^)**	*λ_A_*/*λ_M_*	*A*	Vascularization
**0**	**0**	**0**	**−7.54×10^−1^**	Moderate	**0**	**0**	**−7.54×10^−1^**	Moderate
**3**	**3.14×10^−2^**	**2.55×10^−2^**	**−7.10×10^−1^**	Moderate	**2.04×10^−1^**	**7.76×10^−2^**	**−6.18×10^−1^**	Moderate
**30**	**1.48×10^−1^**	**1.20×10^−1^**	**−5.43×10^−1^**	Moderate	**8.25×10^−1^**	**3.13×10^−1^**	**−2.05×10^−1^**	Moderate
**300**	**4.65×10^−1^**	**2.77×10^−1^**	**−9.30×10^−2^**	Moderate	**1.44×10^0^**	**5.45×10^−1^**	**2.019×10^−1^**	Low

The model classification of tumor vascularization (see **Methods**) for MiaPaCa-2 and S2-VP10 tumors is then based on *λ_A_*/*λ_M_* and parameter *A* with *B* = 0.43.

**Figure 3:**
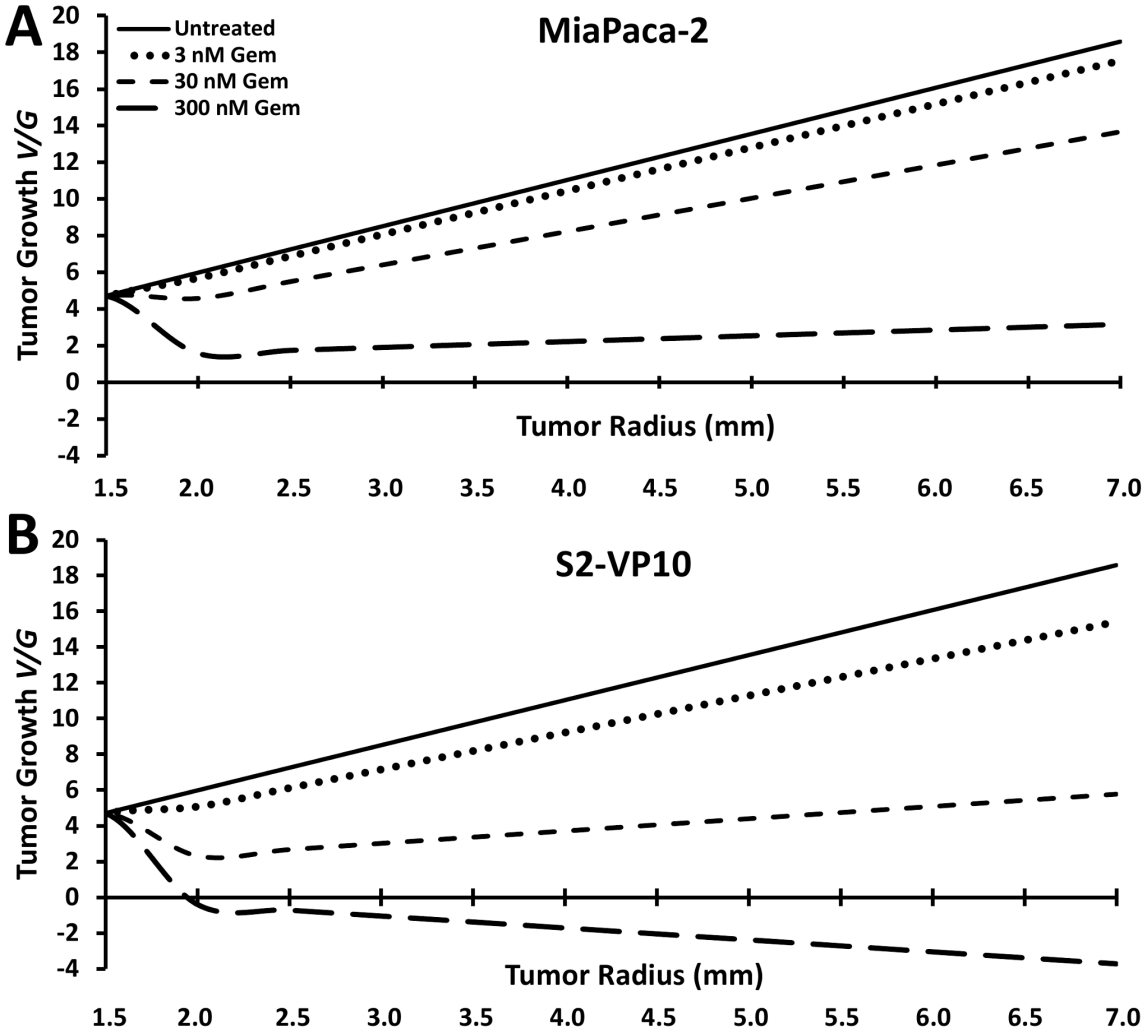
Tumor growth (*V/G*) predicted by the mathematical model. Simulated tumor growth (**Eq. 7**) for radially symmetric (**A**) MiaPaCa-2 and (**B**) S2-VP10 *in-vivo* tumors after treatment beginning when radius *R* = 1.5 mm, taking into consideration diffusion gradients in 3D and a moderate extent of vascularization.

We modeled the tumor growth from the cytotoxic *in-vitro* data using **Eq.7**. **Figure 3A** shows the simulated growth for the MiaPaCa-2 after the beginning of treatment. The model results, which take into account the diffusion of cell substrates from the vasculature in the 3D tissue, demonstrate that this growth is positive regardless of drug concentration. This outcome is contrary to the *in vitro* observations (**Figure 1**), in which cells are treated under optimal exposure in monolayer. The model results further show that the overall growth decreases for higher gemcitabine concentrations. For S2-VP10 the mathematical model shows that the simulated growth is positive except for the 300 nM drug concentration (**Figure 3B**). As with the MiaPaCa-2, the growth decreases at higher gemcitabine concentrations. Reflecting the *in-vitro* data used for parameter calibration, the model results suggest that the growth of S2-VP10 is marginally more sensitive to gemcitabine compared to the MiaPaCa-2.

### *In-Vivo* Orthotopic Tumor Growth

To validate the results from the mathematical model, we evaluated the *in-vivo* efficacy of a weekly treatment of gemcitabine in SCID mice (50 mg/kg mouse) with orthotopically implanted MiaPaCa-2 and S2-VP10 tumors. Tumor growth was examined longitudinally via bioluminescent imaging. Since cell number and bioluminescent light emission are linearly proportional [34], emission signals were used to quantify approximate cell numbers. Tumor radius was calculated from the bioluminescent data assuming radial symmetry, which was converted to a cell number using data on cell radius and packing density (**Supplement**). From H&E (hematoxylin and eosin) staining, cell radii for MiaPaCa-2 and S2-VP10 cells were determined to be ∼9 and ∼7 µm, respectively, and a packing factor for both cell types was determined to be ∼0.761. This packing density was reasonable based on densities as high as 0.813 using a simulation of congruent circles [35], and demonstrated simulation of packing fractions as high as 0.74 [36]. The AUC (area-under-the-curve) for the weekly treatment was ∼337 µg hr/ml; for comparison, gemcitabine concentration exposure in a 24 hour period *in-vitro* was ∼7,600 nM, representing a dosage >25× the maximum *in-vitro*.

**Figure 4:**
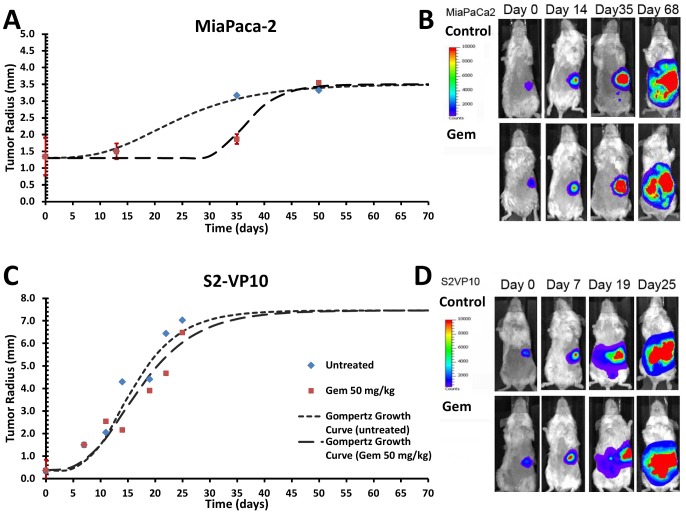
Measurement of tumor radii from bioluminescent imaging of untreated and gemcitabine-treated SCID mice. Gompertz growth curves were fitted to these data to illustrate the tumor growth. (**A**) MiaPaCa-2 radii and fitting to Gompertz equations 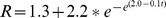 (untreated) and 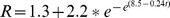 (treated). (**B**) Bioluminescence signal shown for representative mice with S2-VP20 tumors. (**C**) S2-VP10 radii and fitting to Gompertz equations 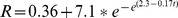 (untreated) and 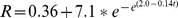 (treated). (**D**) Bioluminescence signal shown for representative mice with S2-VP20 tumors. Error bars in (A) and (C) correspond to standard error of the mean.

Median MiaPaCa-2 tumor radius of the untreated mice increased from 1.3 to ∼3.3 mm (150%) during the observation period (**Figure 4A**); the median radius of gemcitabine-treated tumors increased from 1.3 to ∼3.5 mm (170%) in these mice. Treated tumor radius was not significantly different than the untreated by day 50. Gompertz growth curves [37] of the form 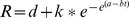 can be fitted to these data showing that treatment with gemcitabine delayed the growth by ∼3 weeks, but did not eradicate the tumor (**Figure 4A**). All of the mice in both groups did not survive beyond day 68 (**Figure 4B**). In comparison, the data for **Figure 4C** indicates that untreated S2-VP10 tumor radius increased from 0.36 to ∼7.0 mm (1840%). Tumor radius in gemcitabine-treated mice increased from 0.36 to ∼6.5 mm (1700%, or 10× that of the MiaPaca-2). Untreated growth was not significantly different than treated by day 25. Comparing untreated and treated growth to fitted Gompertz growth curves shows that the tumors were essentially unaffected by the treatment. All of the mice in both groups did not survive beyond day 25 (**Figure 4D**).

The mathematical modeling predicted that MiaPaCa-2 and S2-VP10 tumors would be resistant to gemcitabine by taking into consideration diffusion gradients in 3D and a moderate extent of vascularization. Except for the 300 nM drug treatment of S2-VP10, this prediction is in agreement with the *in-vivo* data and in contrast to the *in-vitro* cytotoxicity results.

## Discussion

It has been hypothesized that nearly all cancers develop a common set of basic characteristics, namely self-sufficiency in growth signals, insensitivity to anti-growth signals, evasion of apoptosis, limitless replicative potential, sustained angiogenesis, and tissue invasion and metastasis [38]. By focusing on these common elements, mathematical modeling may provide insight into tumor growth and drug response in the 3D *in-vivo* environment. In large scaled systems, continuum methods treat the tumor as a collection of tissue, where densities or volume fractions of cells are described utilizing partial-differential and integro-differential equations [23]. Model variables may include cell volume fractions and cell substrate concentrations, such as oxygen and glucose. These models use parameters that may be measurable from laboratory experiments [39].

The *in-vitro* cytotoxicity experiments demonstrated varying sensitivity between MiaPaCa-2 and S2-VP10 tumor cells to gemcitabine, with the latter exhibiting higher sensitivity. This is consistent with *in-vitro* results by DeRosier et al [40], which showed moderate resistance of MiaPaCa-2 cells to gemcitabine (100% viability at 3 nM; 78% viability at 30 nM), and higher sensitivity of S2-VP10 cells (77% viability at 3 nM; 33% viability at 30 nM). Based on these results, it would be reasonable to expect that treatment with gemcitabine *in-vivo* should demonstrate some efficacy, especially since the highest *in-vitro* experimental gemcitabine AUC at a concentration of 300 nM was only 1.90 µg-hr/ml compared with the *in-vivo* AUC of 337 µg-hr/ml.

Taking into account the diffusion of cell substrates from an impaired vasculature in the 3D tissue (**Eq.1** **,** **Methods**), the mathematical model predicted different outcomes using the same *in-vitro* data. Before simulating the tumor growth using the model, several parameters had to be determined from the *in-vitro* experiments. The extent of vascularization was estimated by observing and calculating the MVD ratio from pancreatic tumor histology, finding that the tumor MVD was significantly lower than that in normal tissue. This is in accordance with observations that lower cell oxygen consumption rates in solid tumors and, in particular, that pancreatic tumor cells are known to tolerate hypoxic conditions and are therefore more resistant to apoptosis [11]–[12]. Lower MVD in tumors has been found in prostate carcinoma, 29% of normal in lung carcinoma, and 78% of normal in glioblastoma [41]. In pancreatic cancer, a decrease in the blood volume fraction has been shown in MRI images as the tumor area increases [42], and a decrease in MVD has been observed with increasing tumor grade [43]. Measuring values of *λ_A_*/*λ_M_* and the parameter *A*, we applied the mathematical model to calculate the rate of growth of these tumors, finding that a positive rate of growth is predicted for all cases but one *in-vivo* (**Figure 3**). By extracting mathematical model parameter values for proliferation and death from monolayer *in-vitro* cytotoxicity experiments, and simulating the effects of spatial diffusion which depend on the extent of vascularization, the model was able to reasonably predict the drug response *in-vivo*.

In agreement with clinical outcomes, the *in-vivo* results demonstrated that gemcitabine had no end effect on the growth of either tumor type and that it did not significantly affect the mortality rate compared to control. We note that the *in-vivo* AUC (337 µg-hr/ml) was at least two orders of magnitude larger than the *in-vitro* AUC (1.90 µg-hr/ml). The only impact the gemcitabine treatment showed was an apparent 3 week delay in the progression of the MiaPaCa-2 tumors (**Figure 4A**), contrary to the *in-vitro* cytotoxicity results (**Figure 1**). The mathematical model reflected this behavior to some extent; the simulated tumor growth (**Figure 3A** **)** decreased between untreated and treated MiaPaCa-2 tumors. Further, the *in-vivo* measurements (**Figure 4**) showed S2-VP10 tumor radii in treated mice to be essentially the same as the untreated, in contrast to the *in-vitro* results (**Figure 1**), indicating higher drug sensitivity of S2-VP10 compared to the MiaPaCa-2 cells. Although the mathematical model also showed non-decreasing S2-VP10 growth for the 3 and 30 nM drug concentrations (**Figure 3B**), it did incorrectly suggest slight tumor regression at the highest dosage (300 nM) as well as a more discernible separation between treated and untreated cases. In comparison, the *in-vitro* results at this dosage would suggest a much more favorable response than that of the simulation. Nevertheless, we believe that the model accuracy can be improved by integration of further biological details currently omitted, e.g., desmoplasia and autophagy that are well known to characterize pancreatic tumors [11]–[12], [44]. Additional bioluminescent imaging beyond once per week may also help to compare the experimental and theoretical tumor responses.

The mathematical model predicted the general response of the tumors to treatment by indicating positive growth *in-vivo* by taking into account the diffusion of cell substrates (e.g., oxygen) as would occur in 3D. In contrast, *in-vitro* monolayer experiments may fail to predict *in-vivo* treatment outcomes in part due to culture conditions representing a single the cell layer closest to the vasculature *in-vivo*, which exposes cells to maximal oxygen/nutrients and drug. Further, ambient oxygen concentration *in-vitro* would prevent cells from becoming quiescent and thus maintains drug sensitivity, contrary to what would occur with cells in tumors *in-vivo* experiencing heterogeneous oxygenation. Using both experiments and computational modeling, it has been shown that 3D cell culture models *in-vitro* demonstrate significantly increased survival and drug resistance over monolayers (e.g., [33] and references therein). As such, parameter values for the mathematical model may be refined from measurements of 3D cell cultures *in vitro*.

Since the model represents tumor growth based on physical principles such as conservation of mass and transport of diffusible substances, the model as applied here is not specifically tailored to pancreatic cancers. As such, the model could be applied to simulate the growth of other solid cancers, e.g., as has been done previously for brain tumors [45]–[46] and lymphoma [47]. Particular predictions of treatment response would depend on the values of the parameters measured from *in-vitro* and vascularization data from these other tumors.

This study demonstrated the feasibility of predicting overall drug treatment effectiveness in an *in-vivo* orthotopic pancreatic tumor model using a mathematical model with parameters mainly set from *in-vitro* data. We apply a simplified mathematical model using a minimum set of parameters to predict the tumor growth. More biologically-complete models might exhibit better predictability as well as broaden the information gained from pharmacokinetic measurements; setting additional parameters would necessitate expanded experimental measurements and increase the model complexity. Our integrated experimental/computational approach may also aid in understanding *in-vivo* experimental and clinical observations which contradict *in-vitro* results focusing mainly on intrinsic resistance mechanisms. The quantification and prediction of treatment response by considering individual tumor phenotypes opens the possibility to design and uniquely strategize innovative targeted treatment experiments. For example, cells extracted from patient biopsies could be cultured *in-vitro* and assessed for cytotoxicity with various drug types and combinations to determine values for the model parameters for apoptosis *λ_A_* and mitosis *λ_M_*, and thus to calculate the strength of apoptosis *A*. The extent of vascularization *B* could be measured from biopsy histology stained for a vascularization marker (e.g., CD31). The model could then be applied to simulate the tumor growth under various treatment scenarios (varying drug concentration and dosages) to assess possible performance *in-vivo*. In this way, mathematical modeling may help to bridge the gap between *in-vitro* and *in-vivo* experimental strategies in order to achieve more effective treatment of pancreatic cancer.

## Supporting Information

Figure S1 Histology slides of pancreatic tissue. (**A**) Normal pancreas H&E staining (inset highlights tissue structure); (**B**) Pancreatic S2-VP10 tumor tissue H&E staining (inset highlights a small blood vessel); (**C**) H&E histology slide showing S2-VP10 tumor cells (middle and right) next to normal pancreatic cells (upper left and left); (**D**) Staining for Factor VIII in section with S2-VP10 tumor cells (100×), used to identify vessels (arrows) for calculation of Microvessel Density (MVD) by cross-sectional area within a given ROI.
(TIF)

Figure S2 H&E histology slide of S2-VP10 tumor cells showing an example of a circular region examined to determine the number of cells within the ROI.
(TIF)

Table S1 Sample set of bioluminescent emission data of S2-VP10 *in-vivo* tumor growth at Day 0 and Day 7.
(TIF)

Table S2 List of model parameters and associated biological meaning.
(TIF)

Text S1 Supplemental material.
(DOC)
