# Supplementary figures and images for: Predictive Modeling of In Vivo Response to Gemcitabine in Pancreatic Cancer
Source: PLoS Comput Biol. 2013 Sep 19;9(9):e1003231. doi: 10.1371/journal.pcbi.1003231 (PMC3777914; doi:10.1371/journal.pcbi.1003231)

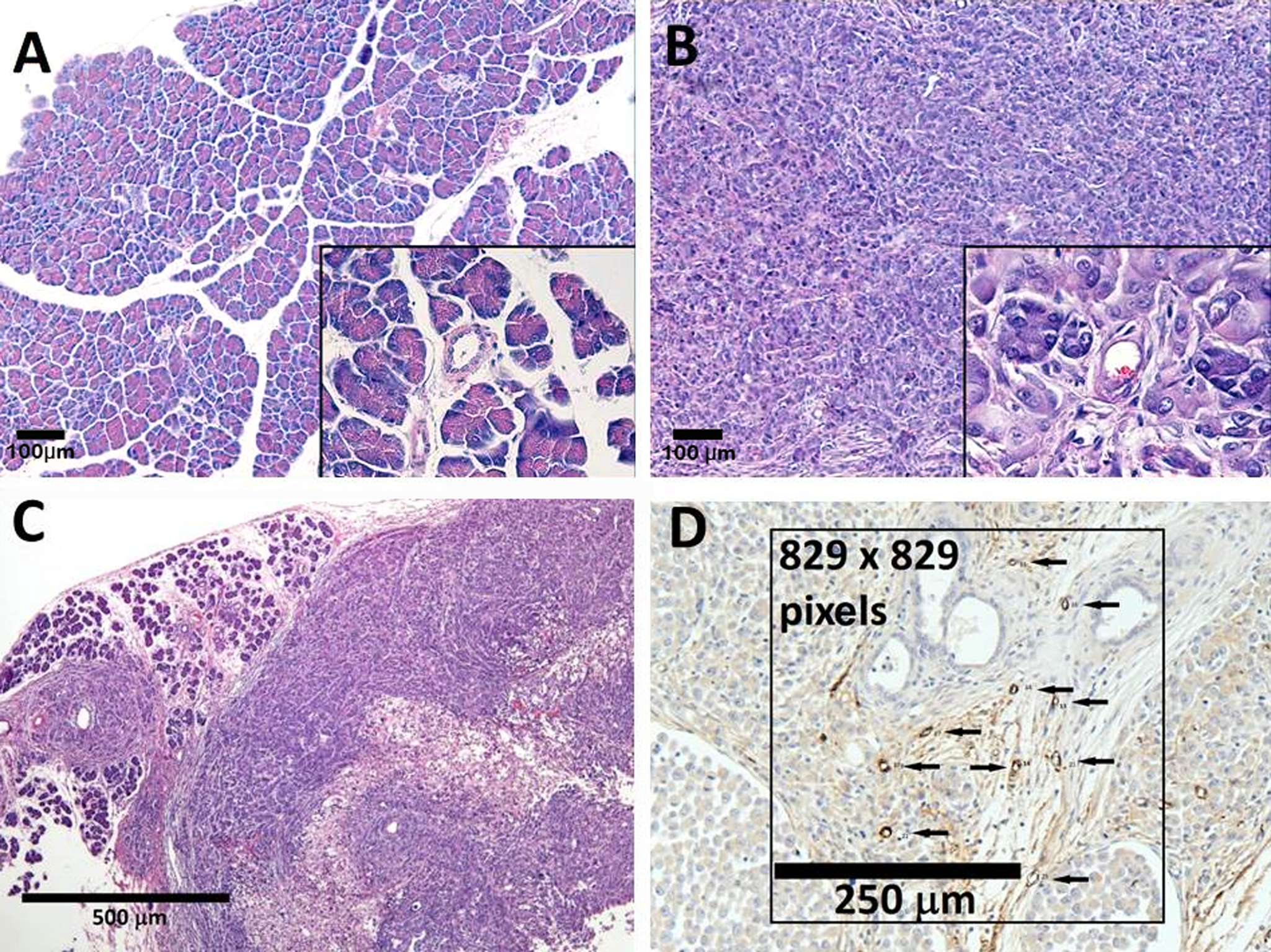

Supplement: Figure S1 — Histology slides of pancreatic tissue. (A) Normal pancreas H&E staining (inset highlights tissue structure); (B) Pancreatic S2-VP10 tumor tissue H&E staining (inset highlights a small blood vessel); (C) H&E histology slide showing S2-VP10 tumor cells (middle and right) next to normal pancreatic cells (upper left and left); (D) Staining for Factor VIII in section with S2-VP10 tumor cells (100×), used to identify vessels (arrows) for calculation of Microvessel Density (MVD) by cross-sectional area within a given ROI. (TIF) [file pcbi.1003231.s001.tif]

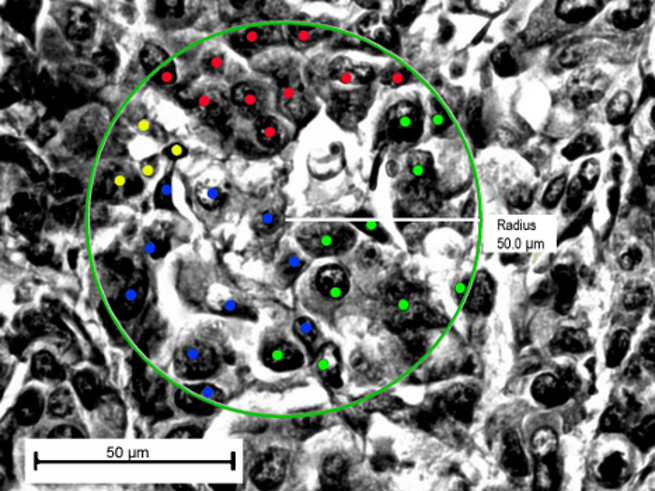

Supplement: Figure S2 — H&E histology slide of S2-VP10 tumor cells showing an example of a circular region examined to determine the number of cells within the ROI. (TIF) [file pcbi.1003231.s002.tif]

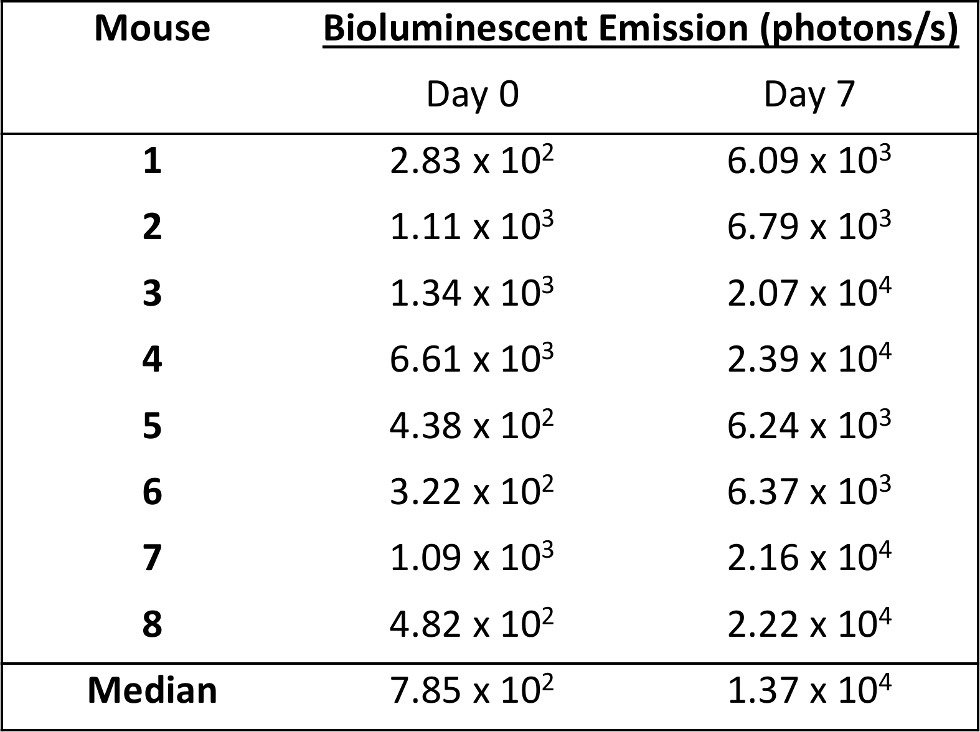

Supplement: Table S1 — Sample set of bioluminescent emission data of S2-VP10 in-vivo tumor growth at Day 0 and Day 7. (TIF) [file pcbi.1003231.s003.tif]

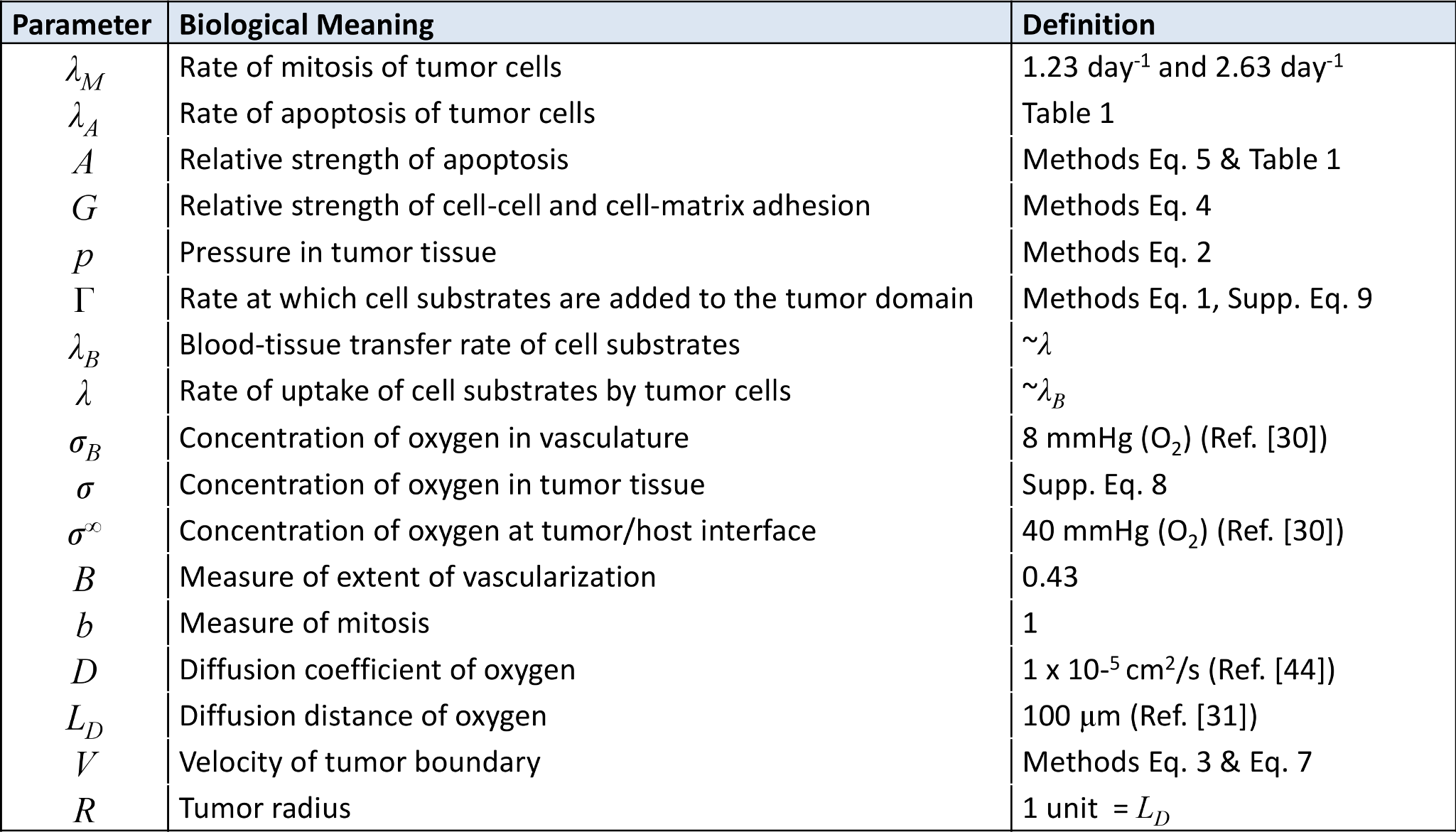

Supplement: Table S2 — List of model parameters and associated biological meaning. (TIF) [file pcbi.1003231.s004.tif]
