# Supplementary material for: Predictive Modeling of In Vivo Response to Gemcitabine in Pancreatic Cancer
Source: PLoS Comput Biol. 2013 Sep 19;9(9):e1003231. doi: 10.1371/journal.pcbi.1003231 (PMC3777914; doi:10.1371/journal.pcbi.1003231)
Supplement: Text S1 — Supplemental material. (DOC) [file pcbi.1003231.s005.doc]

**SUPPLEMENT**

**Microvessel density (MVD)** is a measure of the number of vessels per regions of interest (ROI). Histology slides of both normal pancreas and tumor tissue (**Figure S1**) were examined. The MVD was estimated by evaluating various ROI of pancreatic cells from slides stained for Factor VIII. Microvessels with approximate circular diameters were identified within an ROI at a magnification of either 40x (1.21 μm/pixel) or 100x (0.48 μm/pixel) (**Figure S1D**). The pixels were converted to metric unit lengths and the sum of the microvessel cross-sectional areas was calculated as a ratio to the ROI area, with the resulting microvessel density obtained as a percentage.

**Calculation of tumor cell density.** Within a given circular ROI, the cell number was counted and divided over the ROI area. This cell density was averaged over 5 separate ROIs to yield an estimated packing density of 0.761. **Figure S2** illustrates how cells were identified within an ROI. A sample calculation is as follows:


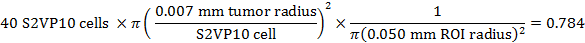


**Calculation of tumor size from bioluminescence.** The bioluminescence signal was quantified from the orthotopic xenografts after injection with luciferin. On Day 0, a specified number of MiaPaCa-2 or S2-VP10 tumor cells were orthotopically injected into the mouse pancreas, followed by luciferin injection and tumor imaging. Day 0 provided a correlation of the bioluminescent signal (photons/s) to the cell concentration, with the lowest concentration of cells providing the most reliable correlation. By day 14 and day 7, the MiaPaCa-2 and S2-VP10 tumor cells, respectively, had both taken root and grown to a typical size of ~1.5 mm radius tumor. A correction factor of the photon/sec/cell correlation was determined to adjust the bioluminescent emission signal to account for the tumor size calculation to the typical diameter of 3 mm. **Table S1** summarizes a sample set of the bioluminescent emission data for untreated mice at day 7 post-orthotopic injection of S2-VP10 tumor cells. The median value was used to calculate the tumor radius. Following is a sample calculation showing how the correction factor and tumor radius were obtained.

Calculation of bioluminescent emission signal correlation to cell concentration on Day 0:


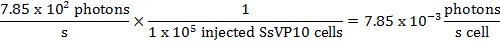


Calculation of correction factor on Day 7 with known tumor diameter of 3 mm:


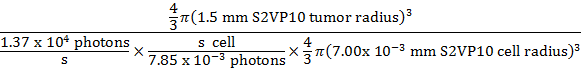


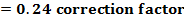


Calculation of tumor radius from median of bioluminescent emission data:


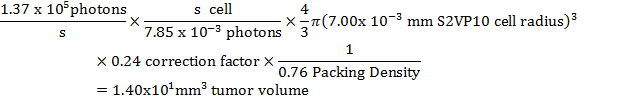


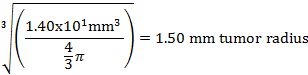


**Mathematical Model**

We summarize the formulation advanced by Cristini et al. (2003) [21] and further developed in [18,22-24] to illustrate the basic model applied in this integrated experimental/computational approach to study pancreatic tumor growth and treatment response. The quasi-steady state diffusion equation for the concentration σ(**x***,t*) of cell substrates is [25-27]:

$0=D\nabla^{2}\sigma+\Gamma$ (8)

where *D* is the diffusion coefficient and Γ is the rate at which cell substrates are added to the tumor domain Ω. The substrates diffuse through the extracellular matrix (ECM), as well as within cells, and are uptaken by tumor cells. The assumption of steady state diffusion [26] is reasonable considering that the rate of diffusion is much smaller (~1 min^-1^) than the rate of cell proliferation (~1 day^-1^). The rate Γ is given by [21]:

$\Gamma=-\lambda_{B}(\sigma-\sigma_{B})-\lambda\sigma$ (9)

This rate includes cell substrates from the vasculature $\lambda_{B}(\sigma-\sigma_{B})$as a source, and the uptake by the cells $[\lambda\sigma]$as a sink. The rate *λ*_B_ is the substrate vasculature-tissue transfer rate, *σ*_B_ is the concentration of substrates in the vasculature, and *λ* is the rate of substrate consumption by tumor cells.

The local rate of tumor volume change$\nabla\cdot\mathbf{u}$ as a function of velocity **u** is obtained by modeling the tumor as an incompressible fluid [21]:

$\nabla\cdot\mathbf{u}=\lambda_{p}$ (10)

The cell proliferation rate *λ_P_* is defined as [21]:

$\lambda_{p}=b\sigma-\lambda_{A}$ (11)

where *b* is a measure of mitosis and
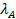
 is the rate of apoptosis, both assumed to be uniform.

The velocity **u** is assumed to follow Darcy’s law [48]:

$\mathbf{u}=-\mu\nabla P$ (12)

where the cell mobility *μ* is assumed constant and $P(\mathbf{x},b)$is the pressure in the tumor domain
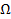
.

The boundary condition for the cell substrate concentration at $\Sigma$ is $(\sigma)_{\Sigma}=\sigma^{\infty}$, where $\sigma^{\infty}$is the uniform concentration outside the tumor volume [21]. The characteristic mitosis rate is a function of this concentration:

*λ*_M_ = *bσ*^∞^ (13)

The tumor pressure is assumed to satisfy the Laplace-Young boundary condition$(P)_{\Sigma}=\gamma\kappa$, where γ is the surface tension related to cell-cell adhesive forces, and _Κ_ is the local total curvature [21].

The tumor velocity $V=\mathbf{n}\cdot(\mathbf{u})_{\sum}$ normal to the tumor boundary is [21]:

$V=-\mu n\cdot(\nabla P)_{\Sigma}$ (14)

The vasculature is assumed to be uniform in this simplified model, and thus the tumor growth is associated with a bulk source of oxygen, nutrients and growth factors [21]. Hence, the growth is limited by the diffusion of cell substrates through the tumor tissue.

An intrinsic length scale in the model is revealed by **Eqs.8-9** [21]:

$L_{D}=D^{1/2}(\lambda_{B}+\lambda)^{-1/2}$ (15)

By non-dimensionalizing the lengths with *L_D_* one obtains from **Eq.12** and the pressure Laplace-Young boundary condition an intrinsic relaxation time scale $\lambda_{R}^{-1}$ corresponding to $\lambda_{R}=\mu\gamma L_{D}^{-3}$ [21]. This rate, associated to relaxation mechanisms such as surface tension γ and cell mobility μ, is used to non-dimensionalize the simulation time.

The dimensionless parameters *A* and *G* and the modified concentration
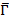
 and pressure $\overline{p}$are specified such that [21]:

$$\sigma=\sigma^{\infty}(1-(1-B)(1-\overline{\Gamma}))$$

$$P=\frac{\gamma}{L_{D}}\left(\overline{p}+(1-\overline{\Gamma})G+AG\frac{x\cdot x}{2d}\right)$$

The dimensionless parameter *B* denoting the extent of vascularization becomes [21]:

$B=\frac{\sigma_{B}}{\sigma^{\infty}}\frac{\lambda_{B}}{\lambda_{B}+\lambda}$ (16)

As described in [21], the non-dimensional equation for
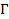
 (**Eq.1**) with boundary condition $(\Gamma)_{\Sigma}=1$ is obtained from **Eqs.9-10** and $(\sigma)_{\Sigma}=\sigma^{\infty}$. The non-dimensional equation for *p* (**Eq.2**) with boundary condition $(p)_{\Sigma}=\kappa-AG\frac{(x\cdot x)_{\Sigma}}{2d}$ is obtained from **Eqs.10-12** and $(P)_{\Sigma}=\gamma\kappa$. The non-dimensional normal velocity *V* of the tumor-host interface (**Eq.3**) is obtained from **Eq.14**.

The bars denoting non-dimensionalized values are omitted in the **Methods**. The main model parameters and their associated biological meaning are summarized in **Table S2**.

**SUPPLEMENTAL REFERENCE**

48. Greenspan HP (1972) Models for the Growth of a Solid Tumor by diffusion. Stud Appl Math LI 4: 317–340.
